# Magnetic Barium Hexaferrite Nanoparticles with Tunable Coercivity as Potential Magnetic Heating Agents

**DOI:** 10.3390/nano14120992

**Published:** 2024-06-07

**Authors:** Diana Zahn, Marco Diegel, Alina Valitova, Jan Dellith, Silvio Dutz

**Affiliations:** 1 Institute of Biomedical Engineering and Informatics (BMTI), Technische Universität Ilmenau, 98693 Ilmenau, Germany; diana.zahn@tu-ilmenau.de (D.Z.); alina.valitova@tu-ilmenau.de (A.V.); 2 Leibniz Institute of Photonic Technology (IPHT), 07745 Jena, Germany; marco.diegel@leibniz-ipht.de (M.D.); jan.dellith@leibniz-ipht.de (J.D.); 3 Faculty of Physical Engineering/Computer Sciences, Leupold Institute for Applied Natural Science (LIAN), Westsächsische Hochschule Zwickau, 08056 Zwickau, Germany

**Keywords:** barium hexaferrite, hard magnetic, magnetic nanoparticles, coercivity, hyperthermia, XRD, SEM, VSM

## Abstract

Using magnetic nanoparticles (MNPs) for extracorporeal heating applications results in higher field strength and, therefore, particles of higher coercivity can be used, compared to intracorporeal applications. In this study, we report the synthesis and characterization of barium hexa-ferrite (BaFe_12_O_19_) nanoparticles as potential particles for magnetic heating. Using a precipitation method followed by high-temperature calcination, we first studied the influence of varied synthesis parameters on the particles’ properties. Second, the iron-to-barium ratio (Fe/Ba = r) was varied between 2 and 12. Vibrating sample magnetometry, scanning electron microscopy and X-ray diffraction were used for characterization. A considerable influence of the calcination temperature (T_cal_) was found on the resulting magnetic properties, with a decrease in coercivity (H_C_) from values above 370 kA/m for T_cal_ = 800–1000 °C to H_C_ = 45–70 kA/m for T_cal_ = 1200 °C. We attribute this drop in H_C_ mainly to the formation of entirely multi-domain particles at high T_cal_. For the varying Fe/Ba ratios, increasing amounts of BaFe_2_O_4_ as an additional phase were detected by XRD in the small r (barium surplus) samples, lowering the particles’ magnetization. A decrease in H_C_ was found in the increased r samples. Crystal size ranged from 47 nm to 240 nm and large agglomerates were seen in SEM images. The reported particles, due to their controllable coercivity, can be a candidate for extracorporeal heating applications in the biomedical or biotechnological field.

## 1. Introduction

One of the major applications for magnetic nanoparticles is their use in medical hyperthermia [1,2,3]. By changing the direction of the particles’ magnetization with an external alternating magnetic field, the particles can produce heat via several mechanisms. In order to efficiently rotate the magnetization and thereby generate the highest possible amount of heat, the strength of the external field has to be adapted to the particles’ coercivity. It is assumed that the field amplitude needs to be two or three times the particles’ coercive field [4]. This limits the coercivity of particles that can be used for medical purposes, due to the safety restrictions for the external magnetic field. When developing magnetic nanoparticles for extracorporeal biomedical heating applications, particles with higher coercivities can be used. Such applications can be the use in thermoresponsive poly-mers [5,6], where magnetic nanoparticles are used to generate the heat necessary for the temperature increase that leads to the deformation of the polymer. Another extracorporeal application is the use as thermal markers on point-of-care diagnostic lateral flow assays, replacing the commonly used color markers (gold nanoparticles that appear as a red line). By using thermal markers, the assays could potentially be used with optically dense and colored sample materials, like blood. To increase the particles’ coercivity, apart from introducing shape anisotropy to iron oxide nanoparticles by synthesizing cubes, rods or discs [7,8,9], or increasing the size of single-domain iron oxide particles [10,11,12], ferrites like cobalt or barium ferrite can be used [13,14,15,16]. These materials exhibit larger anisotropies, which is why the barium hexaferrite BaFe_12_O_19_ is a well-known material for magnetic storage [17] or for permanent magnets [18]. The coercivities of barium hexaferrite nanoparticles used for this purpose, however, are very large, reaching values close to 500 kA/m [19,20], which again would necessitate extremely high external magnetic fields if they are supposed to be used for heating applications. Therefore, we focused on the synthesis and optimization of barium hexaferrite nanoparticles with high magnetizations but coercivities on the lower end of the spectrum possible for this material, to produce particles that are suitable for biomedical heating applications in realistic magnetic fields.

Barium hexaferrite nanoparticles (BaFe-NP) can be synthesized by different routes: hydrothermal synthesis [21,22], sol gel synthesis [23,24], microemulsion [25] or coprecipitation [26,27,28] are reported. Due to its simplicity, fast reaction times and large batch sizes, coprecipitation with a following calcining step was chosen for this study. In the coprecipitation process, several parameters can be varied to influence the particles’ characteristics and a variety of properties is reported for BaFe-NP synthesized via coprecipitation. For the synthesis of monophasic M-type barium hexaferrite, for the ratio of iron to barium ions, usually a value close to the stochiometric ratio of 12 or a slightly iron-deficient ratio < 12 is used [29,30]. Varying Fe/Ba ratios has been investigated by research as well, mostly by changing the ratio towards a barium surplus. However, only few studies are available that report structural and magnetic parameters together, while many only give the phase composition depending on the Fe/Ba ratio or structural characterization [31,32]. Some studies that report magnetic properties for different Fe/Ba ratios come to divergent results. Dudziak et al. [33] found a higher H_C_ with increased Fe/Ba, while Li et al. [34] found only minor changes in H_C_ with a slight decrease in H_C_ for high Fe/Ba ratios. For the calcination duration, most studies choose a short time of 1 to 4 h [35,36,37], which seems sufficient to achieve particles with a suitable magnetic behavior. The majority of coercivities reported for synthesized barium hexaferrite particles are in the range of 200 kA/m to 450 kA/m [27,37,38,39,40,41], as expected for this high anisotropy material. The temperatures used for the calcination of the precursors to form the magnetic hexaferrite phase have been investigated, with different results. In [38], it was reported that coercivity slightly increases from 419 kA/m to 461 kA/m when increasing the temperature from 639 °C to 919 °C. Saturation magnetization in this study was low for the sample calcined at 639 °C, but reached values > 60 Am^2^/kg for higher calcination temperatures. Rashad et al. [42] also found an increasing trend of H_C_ with T_cal_ with H_C_ values of 150 kA/m at 800 °C and 265 kA/m at 1200 °C. The same trend was reported by Lisjak et al. [27], with H_C_ increasing from 95 kA/m at 500 °C to 382 kA/m at 80 °C. However, some authors reported a decrease in H_C_ for high T_cal_. In Yensano et al. [39], H_C_ decreased from 310 kA/m to 191 kA/m when T_cal_ was increased from 800 °C to 1000 °C, and in Janasi et al. [36], H_C_ dropped from 440 kA/m to 162 kA/m with an increase in T_cal_ from 800 °C to 1100 °C. This decrease for high temperatures is often explained by an increase in particle size and therefore a transition from a single-domain to a multi-domain state. The critical diameter for barium hexaferrite for the formation of single domains is reported from several hundreds of nm up to > 1 µm [20,43], indicating that single-domain particles can exist up to rather large particle sizes for this material. 

As mentioned above, we are focused on the synthesis of barium hexaferrite particles that are suitable for ex vivo biomedical heating applications, and therefore a coercivity is needed that is high enough for an open hysteresis cycle producing significant heating power, and low enough to be efficiently heated by the available external magnetic field. At the same time, high magnetizations are needed for a good heating performance. To enable the synthesis of those tunable barium hexaferrite particles, we first varied several synthesis parameters including pH, the temperature of the calcination (T_cal_) and the duration of the calcination (d_cal_). In the second part, the synthesis parameters were constant and the Fe/Ba ratio was varied from 2 to 12. The resulting particles were characterized using vibrating sample magnetometry (VSM) at room temperature, X-ray diffraction (XRD) and scanning electron microscopy (SEM).

## 2. Materials and Methods

### 2.1. Particle Synthesis

Barium ferrite nanoparticles with the composition BaFe_12_O_19_ were synthesized using a coprecipitation technique. Barium nitrate (Ba(NO_3_)_2_), iron(III) nitrate nonahydrate (Fe(NO_3_)_3_ ∙ 9H_2_O) and sodium hydroxide (NaOH) were obtained from Carl Roth GmbH + Co. KG, Karlsruhe, Germany. First, (Ba(NO_3_)_2_) and (Fe(NO_3_)_3_ ∙ 9H_2_O) were dissolved in 100 mL of deionized (DI) water at room temperature. The ratio of Fe/Ba was kept at 11.5 if not otherwise stated. The solution of nitrates was initially stirred for 20 min in deionized water using a magnetic stirrer. Next, a gradual addition of 3M NaOH was performed to adjust the pH and initiate the precipitation process. The final pH value was varied between 8 and 13 by adding different amounts of the NaOH solution using a digital pH meter for control. The resulting suspension was stirred for 30 min after reaching the required pH value. The dark brown precipitate underwent purification, which included repetitive centrifugation (UNIVERSAL 32, Andreas Hettich GmbH & Co. KG, Tuttlingen, Germany) and three washing steps with DI water. Last, the precipitate was dried for 24 h at 40 °C using a drying oven (Heraeus UT 6120, Kendro Laboratory Products GmbH, Langenselbold, Germany).

The dried powder was briefly ground by hand with a glass mortar and subsequently subjected to calcination in a high-temperature furnace (Nabetherm GmbH, Lilienthal, Germany). The samples underwent a single-stage calcination process, with the calcination temperature T_cal_ being varied from 800 °C to 1200 °C and held for durations d_cal_ of 3 h to 24 h. 

The parameters and sample names used are summarized in Table 1. Samples are named according to the used synthesis parameters as d_cal__T_cal__pH.

As a second part of this study, the molar ratio of Fe/Ba (r) was varied between 2 (surplus of barium) and 12 (stochiometric ratio for BaFe_12_O_19_) in steps of r = 2, 5, 6.5, 8, 10, 11.5 and 12. In order to achieve the desired ratio of the precursor ions, the mass of the two nitrates relative to each other was varied while the absolute amount of metal nitrate salts was kept constant at 0.025 mol. The calcination parameters, including the calcination temperature (T_cal_ = 1200 °C), calcination duration (d_cal_ = 3 h), and pH value (pH 12), were maintained constant. 

### 2.2. Particle Characterization

#### 2.2.1. Vibrating Sample Magnetometry (VSM)

The magnetic properties of the calcined powders were investigated at room temperature using a vibrating sample magnetometer (VSM, Model: MSE-EZ9, Microsense, Lowell, MA, USA). For this purpose, approximately 3 to 6 mg of the calcined and hand-ground powder were transferred into a 3D-printed polymeric sample holder, weighed and compressed for immobilization of the particles. 

All hysteresis curves were recorded with a maximum applied field of ±2 T (1591 kA/m) at room temperature with a decreased step size in the low-field region for an accurate determination of the coercivity. Magnetization at 2 T, named M_2T,_ and the coercivity H_C_ were extracted from the hysteresis curves.

#### 2.2.2. X-ray Diffraction (XRD)

Structural properties were investigated with X-ray diffraction (Panalytical X’Pert Pro MPD theta–theta diffractometer, PANalytical, Almelo, The Netherlands) using copper radiation, a parallel mirror and a PIXCEL3D Si detector in 1D mode. The measured 2 theta range was chosen from 20° to 80° with a step size of 0.026° and 1 h measuring time. To suppress most of the Fe fluorescence signal, the lower detector threshold was kept at 47.5%, reducing the background level from approximately 67,000 counts to below 1000 counts and increasing the signal-to-noise ratio from 98 to 151 for the 100% peak. A polycrystalline Si disc was used as a reference sample for determination of the instrumental broadening. Phase analysis, phase quantification and determination of crystallinity and crystal size were carried out with MalvernPanalytical HighScore Plus V.4.9 [44]. For phase identification, the databases ICDD PDF2 from 2001, ICSD from 2008 and 2024 and COD from 2023 were used. Quantification in multiphase samples was carried out using Hideo Toraya’s “Direct Derivation Method” (DDM) [45]. 

Crystallinity determination was carried out by the “flat background method”. The measured scans were compared with the scan of a 100% crystalline M-type barium hexa-ferrite powder reference sample, BaFe_10.3_Co_0.85_Ti_0.45_Sn_0.4_O_19_, prepared by the glass crystallization method. This method is known for preparation of nanoparticles showing a very high crystallinity [46]. The reference sample has a hexagonal lattice like BaFe_12_O_19_ with very similar lattice parameters. The peak shift of the FWHM-value for the 100% peak is below 10%. Due to different sample amounts of the reference and the to-be-investigated samples of this study, and thus different count statistics, every sample scan was adjusted to the reference sample scan with an individual scale factor and offset value, obtained from background line adjustment. In multiphase-samples, crystallinity-values above 100% were calculated, due to the higher number of peaks in these samples. Crystallinity for those samples was set to 100%. None of the reported samples showed any signal arising from amorphous components.

Crystallite size and micro strain determination was conducted with quadratic Williamson–Hall plots from Pawley fits for the main BaFe_12_O_19_ phase. Peaks with an intensity below 1% of the highest intensity peak were excluded for the Pawley fit.

#### 2.2.3. Scanning Electron Microscopy (SEM)

Scanning electron microscopical investigations were performed on a Tescan LYRA XMU dual beam system (TESCAN, Brno, Czech Republic). For this purpose, the sample powders were deposited on conductive sticky carbon pads and coated with approximately 8 nm carbon in order to ensure a sufficient electrical conductivity. The electron energy E_0_ was set to 15 keV in most cases. Secondary electron (SE) images were taken using an Everhard Thornley-type detector. For selected samples, a YAG-type detector for backscattered electrons (BSEs) was used to emphasize the topography and to suppress partially occurring charging effects. Moreover, the BSE images provide an atomic number or compositional contrast to visualize potential material inhomogeneities. Such inhomogeneities were partially investigated in more detail by X-ray element mappings using a Bruker XFlash 7030 EDX system. 

## 3. Results and Discussion

### 3.1. Variation of Synthesis Parameters

In the first part of this study, the synthesis parameters T_cal_, d_cal_ and pH were varied. The results of the structural characterization using XRD and magnetic characterization with VSM can be seen in Table 2. All particles in this series of experiments had an Fe/Ba ratio (r) of 11.5.

#### 3.1.1. Phase Composition

In all the samples, M-type barium hexaferrite BaFe_12_O_19_ was confirmed as the main phase, with amounts ranging from 87.1 wt% to 100 wt%. In some samples, especially those synthesized at pH 8, as well as in one sample synthesized with pH 12 (9_800_12), hematite α-Fe_2_O_3_ was found as an additional phase, with a content of up to 10.5 wt%. The formation of hematite as an intermediate phase in the synthesis of BaFe_12_O_19_ is well known [20]. Hematite is assumed to react with Ba(OH) to form hexaferrite, but in the case of an incomplete reaction due to low temperatures or low pH [32,40,47], hematite remains in the final particles. Apart from this iron oxide, BaFe_2_O_4_ was found in two samples (3_1200_12 and 12_800_12). The formation of this monoferrite as an intermediate is also known for barium hexaferrite synthesis [20,31,32]. BaCO_3_ was found in the literature to form for synthesis under air starting with metal nitrates as educts, which is followed by the reaction of BaCO_3_ with hematite to BaFe_2_O_4_ [27]. Other phases found were the W-type hexaferrite BaFe_18_O_27_ in sample 3_800_12 and FeNaO_2_ in sample 3_800_13. A small amount of barium hydroxide, Ba(OH)_2_, was detected for sample 3_1200_8.

Crystallinity, as extracted from the XRD measurements, was very high for all the samples, ranging from 91.7% to 100%, and will be discussed in more detail in the following section, where the synthesis parameters are considered individually.

#### 3.1.2. Influence of the Duration of Calcination d_cal_

**Figure 1:**
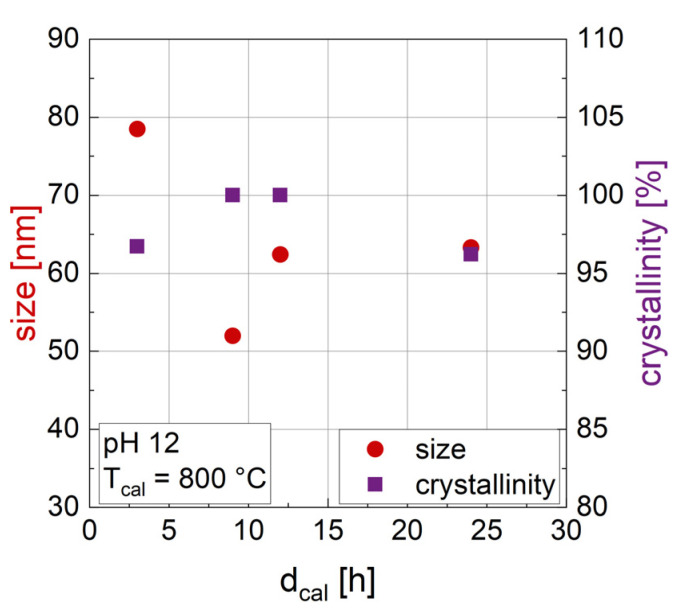
Crystal size derived from the hexaferrite phase in XRD (red) and crystallinity (purple) of BaFe-NP synthesized with varying d_cal_. pH was kept constant at a value of 12 and T_cal_ was 800 °C.

Changing the duration of calcination (d_cal_) (Figure 1) does not result in a clear trend in the crystallite size of the particles, as determined by XRD. Crystallinity is also not significantly affected by d_cal_, with values above 95% for all samples shown in Figure 1. The rather random relation of crystal size with d_cal_ indicates that during calcination (with T_cal_ = 800 °C) a rearrangement by diffusion within the crystal lattice takes place to form hexa-ferrite, but crystals do not grow in size during calcination. Therefore, crystallite size seems to be mainly defined by the precipitation step of the synthesis and temperature of the calcination, which will be discussed below. In accordance with our results, Gjørup et al. [48] found almost stable crystal sizes for durations of calcination from 1 to 16 h at a temperature of 800 °C, but a change in size depending on temperature. Please note that the size discussed here is the crystal size, which does not allow any statement on the size of the multi-crystalline particles that might be forming. The dependence on calcination time (3 h vs. 9 h) was confirmed for another calcination temperature (1200 °C) and different pH (8, 10 and 13) as well where again no considerable trend of crystal size with varied d_cal_ was found. Looking at the phase composition in Table 2, pure M-type barium hexaferrite was confirmed in XRD for d_cal_ = 24 h (sample 24_800_12), which could indicate that longer calcination times are necessary for the formation of hexaferrite. However, pure hexaferrite was also confirmed for other synthesis parameters with a shorter d_cal_ of 3 h and with higher T_cal_ and/or higher pH, indicating that the combination of parameters is important, not the duration of calcination alone.

**Figure 2:**
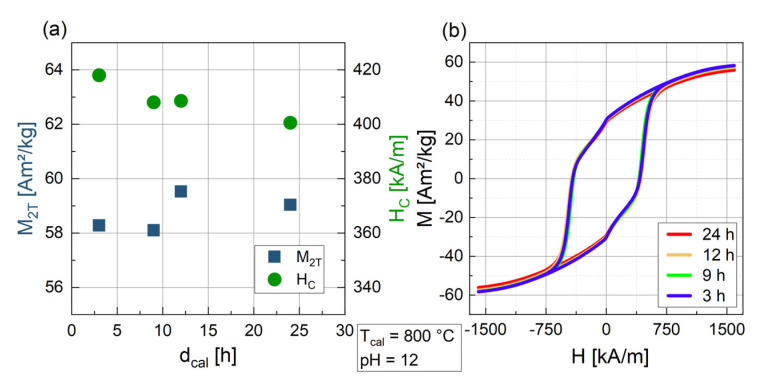
Magnetic parameters M_2T_ and H_C_ for BaFe-NP synthesized with varying d_cal_ at pH 12 and with T_cal_ = 800 °C (**a**) as extracted from hysteresis curves as seen in (**b**). Different d_cal_ are indicated by color coding in (**b**).

Particles with different d_cal_ were analyzed with VSM to determine their magnetic properties (M_2T_ and H_C_) at room temperature and with a maximum field of 2 T. Looking at the influence of d_cal_ on the magnetic properties (Figure 2), no considerable dependence is found. H_C_ slightly decreases from 418 kA/m to 400 kA/m while M_2T_ is almost constant, ranging from 58.1 to 59.5 Am^2^/kg. As mentioned above, d_cal_ was also evaluated for T_cal_ = 1200 °C and for other pH values (8, 10 and 13), where again no change in magnetization was found and only minor variations in coercivity occurred. This again indicates that after 3 h of calcination, no significant changes within the crystal structure are taking place. For this reason, 3 h was chosen as the calcination time for the following experiments.

#### 3.1.3. Influence of the Temperature of Calcination (T_cal_)

**Figure 3:**
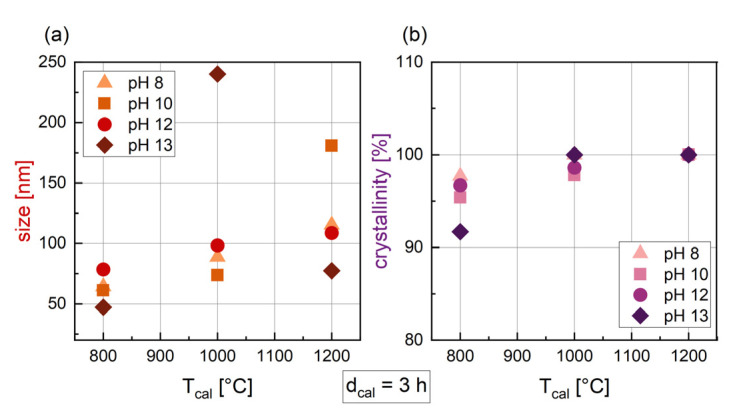
Crystal size derived from the hexaferrite phase in XRD (**a**) and crystallinity (**b**) for BaFe-NP synthesized with varying T_cal_ and d_cal_ = 3 h. Data are shown for different pH values during synthesis.

After changing the calcination temperature from 800 to 1200 °C, a small increase in crystal size can be seen for synthesis with pH 8 to 12 (Figure 3a), while for pH 13 a maximum size at T_cal_ = 1000 °C was found. In general, the size of particles synthesized with varying T_cal_ and with d_cal_ = 3 h ranges from 47.3 nm to 240.1 nm. An increase in crystal size with rising temperature can be confirmed by the literature, with several authors reporting the same trend. Yenaso et al. [39] found an increase in size from 60 nm to 77 nm when increasing the temperature from 800 °C to 1000 °C with a pH of 14. Hu et al. [40] reported an even more pronounced increase in size from 63 nm at 800 °C to 153 nm at 1100 °C (pH = 9 and d_cal_ = 2 h). For crystallinity (Figure 3b), a slightly increasing trend can be assumed, with higher T_cal_ leading to higher crystallinities. For T_cal_ = 1200 °C, all the samples are 100% crystalline.

**Figure 4:**
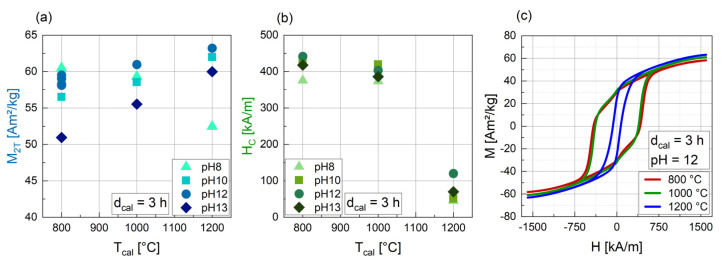
Magnetic parameters M_2T_ (**a**) and H_C_ (**b**) for BaFe-NP synthesized with varying T_cal_ at varying pH values and at d_cal_ = 3 h. Related hysteresis curves for pH 12 are shown in (**c**).

The calcination temperature has a significant influence on the magnetic parameters (Figure 4). Apart from the samples synthesized at pH 8, all the particles show an almost linear increase in M_2T_ with increasing T_cal_. An increase in magnetization with T_cal_ was also found by Hu et al. [40] and Kwak et al. [41]. H_C_ is nearly the same for all pH values at 800 °C and 1000 °C (373 kA/m to 441 kA/m), but drops significantly to values between 45 and 70 kA/m for T_cal_ = 1200 °C (Figure 4b), which is also evident in the M(H) curves in Figure 4c. In addition, for samples 3_800_12 and 3_1000_12, a “peanut shape” of the M(H) curve with a slight drop after passing a magnetic field of zero is evident. This indicates that these samples contain a hard and a soft magnetic fraction. 

**Figure 5:**
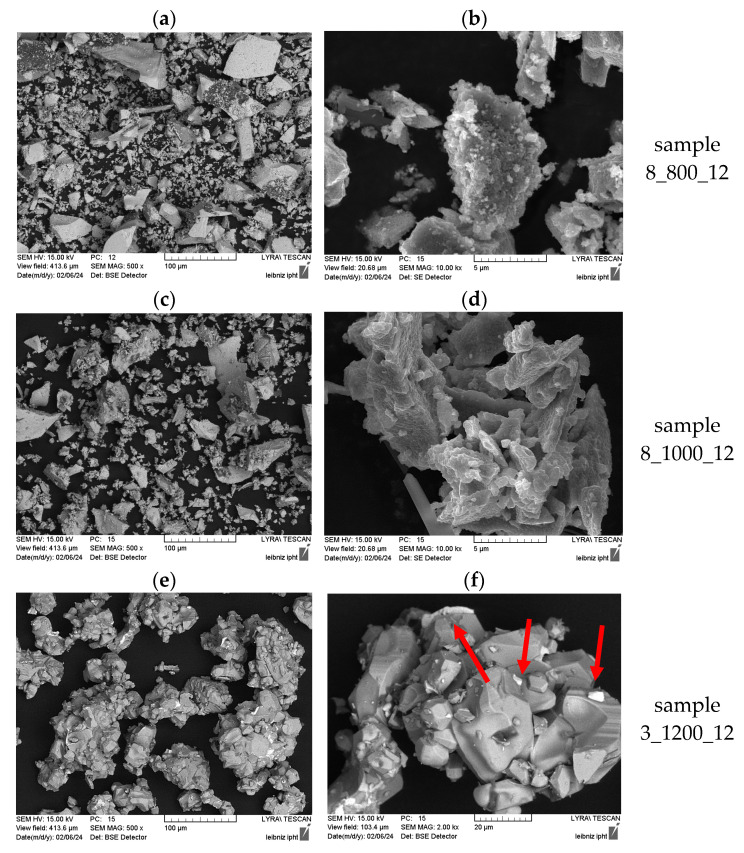
SEM images, partially taken with backscattered electrons (BSEs), for samples calcined at 800 °C (**a**,**b**), 1000 °C (**c**,**d**) and 1200 °C (**e**,**f**). For sample 3_1200_12, bright particles with increased Z-contrast in BSE mode are marked with red arrows.

To correlate the magnetic properties with the structural appearance of the particles, SEM images were taken for samples calcined with 800 °C (3_800_12), 1000 °C (3_1000_12) and 1200 °C (3_1200_12). The SEM images can be seen in Figure 5. The samples calcined at 800 °C and 1000 °C consist of particles and agglomerates of large sizes and with a very broad size distribution. Some particles were found with a “bubbly” surface, showing a rough and slightly porous structure. A fraction of very small particles can be seen in these samples, which might be below the critical size of single-domain particles, which is reported to be from several hundred nm up to 1.3 µm [20,26]. This mixture of very small, single-domain particles with a large coercivity and large particles consisting of multiple magnetic domains, leading to a soft magnetic behavior, can explain the large coercivity of these samples and the “peanut shape” with a slight drop in magnetization around the zero field. For sample 3_1200_12, in contrast, this smallest fraction was not found and the particles are well above the critical size for single domains. The formation of almost entirely multi-domain particles can explain the decreasing coercivity for the samples calcined at 1200 °C. In sample 3_1200_12, some particles appear with a brighter contrast in the BSE (back scattered electron) image (marked with red arrows in Figure 5), what indicates a higher mean atomic number. This could indicate Ba-rich phases, which was confirmed by EDX analysis. 

The crystal size determined with XRD and the size of particles seen in SEM differs significantly. A particle can consist of several crystals; however, the morphology seen in the SEM images suggests at least partially (strongly agglomerated) monocrystalline particles. Crystal size determination by XRD is reliable for crystal sizes up to appr. 60 nm for a parallel beam geometry. For larger crystallites up to 100 nm and above, a SEM inspection as a control measurement is beneficial. With Cu-Kα_1,2_-radiation, the maximum size measurable by XRD is limited to 100–200 nm [49]. In addition, only the main phase was used for the Williamson–Hall plot, and other phases in the sample were not included.

Due to this study being focused on the optimization of BaFe-NP for magnetic heating and therefore H_C_ in the lower range of possible values for BaFe-NP being favored, T_cal_ was chosen to be 1200 °C for the following experiments. 

#### 3.1.4. Influence of the pH

**Figure 6:**
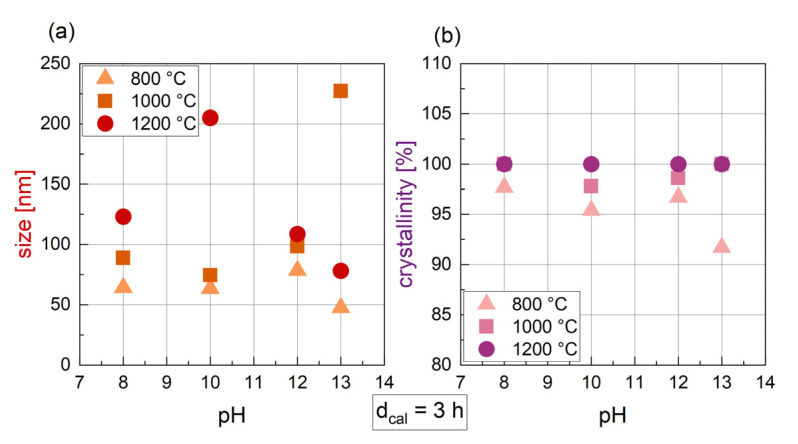
Crystal size derived from the hexaferrite phase in XRD (**a**) and crystallinity (**b**) for BaFe-NP synthesized with varying pH. Data are shown for different T_cal_ (color-coded) and d_cal_ = 3 h.

Varying the pH that is reached after adding the alkaline medium to the metal nitrate solution, no clear trend is evident for the crystal size (Figure 6a). For T_cal_ = 1000 °C, the crystal size increases considerably for the highest pH used, while for T_cal_ = 1200 °C, a pH of 10 leads to the biggest crystal size. Considering the crystallinity (Figure 6b), crystallinity slightly drops for pH 13 and a low T_cal_ of 800 °C. For higher T_cal_ (1000 °C and 1200 °C), a change in the pH does not influence the crystallinity. In addition, the pH seems to have an influence on the phases that are formed. As already discussed, for pH 8, hematite was found for all T_cal_ (see Table 2). Hematite as a remaining intermediate phase was also found in [50] for pH 8 and 10 as well as BaFe_2_O_4_ for pH 10. However, the authors found an increase in size and crystallinity with increasing pH. Yensano and al. [39] found hematite and BaFe_2_O_4_ for low pH synthesis as well.

**Figure 7:**
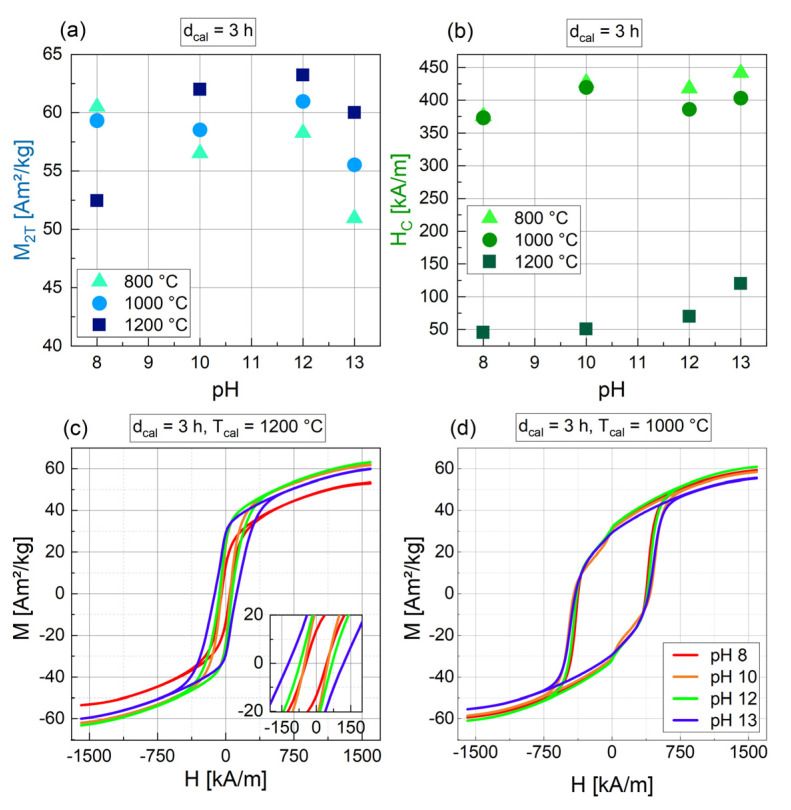
Magnetic parameters M_2T_ (**a**) and H_C_ (**b**) for BaFe-NP synthesized with varying pH at three different temperatures T_cal_ (color-coded) and with d_cal_ = 3 h. Related hysteresis curves for T_cal_ = 1200 °C (**c**) and for T_cal_ = 1000 °C (**d**) are shown below with the pH indicated by color coding.

Depending on the calcination temperature used after the precipitation step, different trends can be seen in the magnetic characterization for different pH values (Figure 7). For T_cal_ = 800 °C and 1000 °C, M_2T_ is rather constant for pH 8 to 12 and drops slightly for pH 13 (Figure 7a). H_C_ for the same T_cal_ is high, with values of 373 kA/m to 441 kA/m (Figure 7b). For T_cal_ = 1200 °C, in contrast, a pH of 8 leads to a low M_2T_ of 52 Am^2^/kg, while a higher pH from 10 to 13 enables high M_2T_ with values ranging from 60 to 63 Am^2^/kg (Figure 7a). The related H_C_ for T_cal_ = 1200 °C is much lower compared to T_cal_ = 800 or 1000 °C, with values from 45 kA/m to 120 kA/m and a slightly increasing trend with increasing pH. The relation between pH and H_C_ is clearly evident in the hysteresis curves. For T_cal_ = 1200 °C (Figure 7c), the hysteresis curves are rather narrow with a bigger H_C_ for a higher pH, while for T_cal_ = 1000 °C (Figure 7d), the hysteresis curves are very broad and H_C_ is nearly constant. 

In conclusion, the pH for the reported synthesis method should be chosen to be higher than 8, due to the tendency of hematite formation for pH = 8. Further, the pH chosen should depend on the coercivity needed, since a small influence of pH on H_C_ was found (for T_cal_ = 1200 °C). If a high magnetization is needed, pH = 13 should be avoided, due to the slight drop in M_2T_ for this pH.

#### 3.1.5. Relation of Synthesis Parameters and Crystal Size

To summarize, the XRD-derived crystal size for particles synthesized with different synthesis parameters ranges from 47.3 nm to 240.1 nm, while the particle size determined from SEM can be estimated with a very broad size distribution, ranging from appr. 1 µm to several tenth of microns. Large agglomerates are seen, along with a variety in the particle shapes and morphologies. This indicates that the particles are polycrystalline, since the crystal size seen in the XRD is much smaller than the physical particle size. As discussed above, the habitus of the particles seen in the SEM points towards agglomerated single crystals with sizes way above the XRD-derived crystal size. However, the size estimated by XRD has to be considered with care, since only the peaks of the main component, the hexaferrite phase, were included into the fit for the Williamson–Hall plot, which was used for size evaluation. The influence of the additional phases, therefore, is not taken into account for the crystal size. Also, as already discussed above, large crystals above appr. 100–200 nm in size do not contribute to the size evaluation. It can be assumed that the XRD crystal size produces a downward estimate of the actual crystal size.

**Figure 8:**
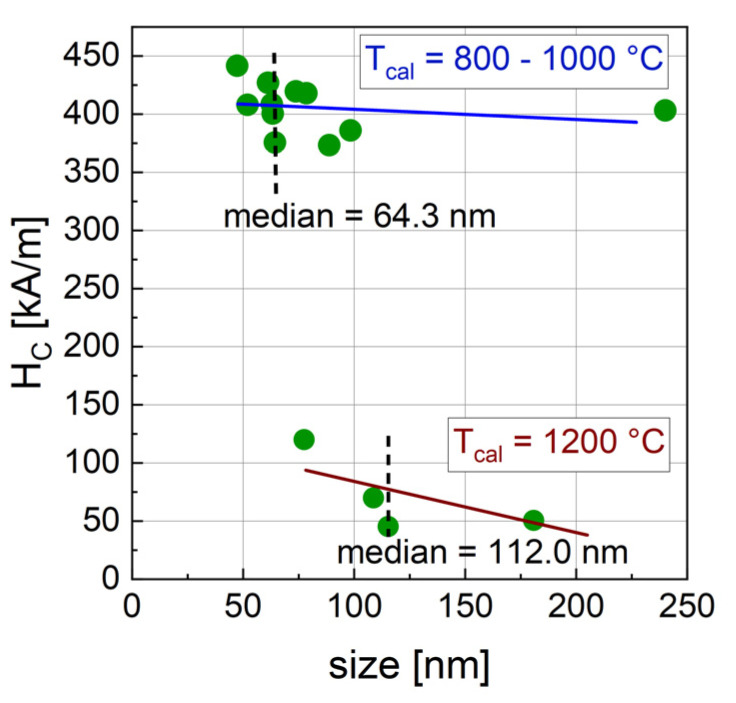
Relation of H_C_ with crystal size derived from the hexaferrite phase in XRD of BaFe-NP with varied synthesis parameters. The ensemble in the upper part was synthesized with T_cal_ = 800 °C and 1000 °C; the ensemble at the bottom was synthesized with T_cal_ = 1200 °C. The dashed lines indicate a calculated median for the two ensembles. The solid lines indicate a linear fit for the data points of the respective ensemble.

For the correlation of H_C_ with crystal size, two populations of particles are found (Figure 8). The particles synthesized with a T_cal_ of 800 or 1000 °C exhibit high coercivities that show only a slight decrease with increasing crystal size and the majority of this population has small crystal sizes ranging from 47.3 nm to 98.4 nm. The crystal size of one sample (3_1000_13) was determined to be 240.7 nm, which is considerably larger compared to the other samples in this ensemble. This sample was found to be monophasic barium hexaferrite and no apparent reason for its increased size was found, wherefore it might be handled as an outlier. Including this potential outlier in the analysis, the median for this ensemble is 64.3 nm. The particles synthesized with T_cal_ = 1200 °C have lower H_C_ and crystal sizes between 77.4 nm and 180.8 nm (median = 112.0 nm) and a slight decreasing trend of H_C_ with size can be assumed. A considerable change in anisotropy has to be assumed when T_cal_ is increased from 1000 to 1200 °C. For the magnetic structure of the particles, the critical size (d_crit_) below which single-domain particles are reported for barium hexaferrite ranges from 100 to 300 nm [43] up to 1.3 µm [20], with the majority of the literature pointing towards a rather large d_crit_. Our particles show fractions around and above the reported d_crit_. For low T_cal_, a fraction of very small particles was seen, which might explain the hard magnetic behavior with high coercivities due to single-domain formation. As discussed above, for high T_cal_, the formation of almost entirely large multi-domain particles is assumed, matching the decreased coercivity. Within the multi-domain range, we see a slight decrease in H_C_ with increasing crystal size. It was shown that magnetic domains are larger in size for larger crystal sizes [51] and that coercivity decreases with increasing domain size [52], explaining the observed magnetic behavior of our particles.

### 3.2. Variation of Fe/Ba Ratio (r)

After evaluating the influence of the synthesis parameters on the resulting BaFe-NP, the ratio of iron to barium ions (r) was varied between 2 (excess Ba^2+^ ions) and 12 (stochio-metric ratio for BaFe_12_O_19_). The synthesis parameters were kept constant with d_cal_ = 3 h, pH = 12 and T_cal_ = 1200 °C. An overview of the results for the particles synthesized with a varying Fe/Ba ratio (r) is given in Table 3.

#### 3.2.1. Phase Composition

**Figure 9:**
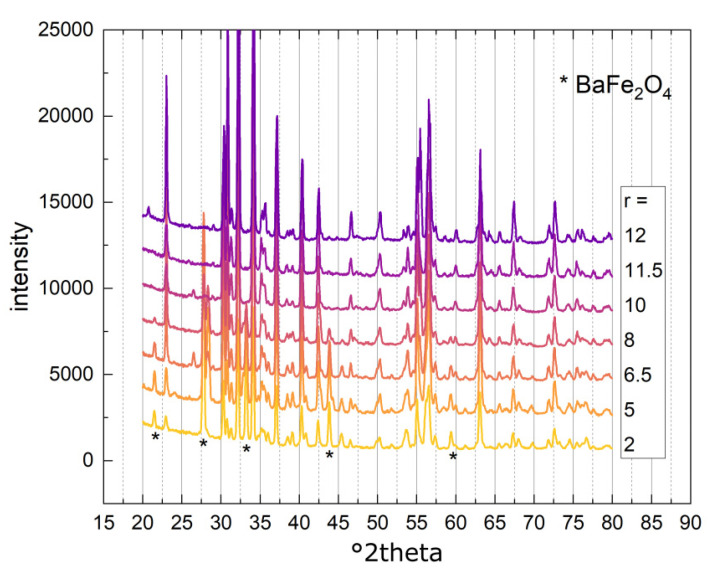
XRD diffractograms of BaFe-NP synthesized with varying Fe/Ba ratio (r). Peaks attributed to BaFe_2_O_4_ are marked with a star. Diffractograms are shifted to higher intensities for better visibility.

Pure M-type hexaferrite was synthesized with r = 11.5, slightly below the stochiometric ratio in BaFe_12_O_19_. For r = 12, only a very small amount of 0.5 wt% of Fe_12_Na_4_O_20_ was detected in addition to the hexaferrite. In the literature, one can find several reports that indicate an iron-deficient ratio is beneficial for the synthesis of the pure M-type hexaferrite phase [29,32,33]. Apart from BaFe_12_O_19_, the barium monoferrite BaFe_2_O_4_ was found in samples with a low r between 2 to 8. Compared to the hexaferrite, this barium-rich phase tends to form when a barium excess is present in the initial solution. As mentioned before, BaFe_2_O_4_ is assumed to be an intermediary in the formation of barium hexaferrite. In the sample with r = 5, small amounts of iron oxide Fe_21.4_O_32_ were detected and two different barium iron oxides were detected for sample r = 10. The XRD diffractograms of all the samples with varied r are seen in Figure 9. The diffractograms mainly show the characteristic peaks for BaFe_12_O_19_, with some peaks assigned to the BaFe_2_O_4_ phase (indicated by stars in Figure 9), which decrease in intensity with an increase in the Fe/Ba ratio. 

**Figure 10:**
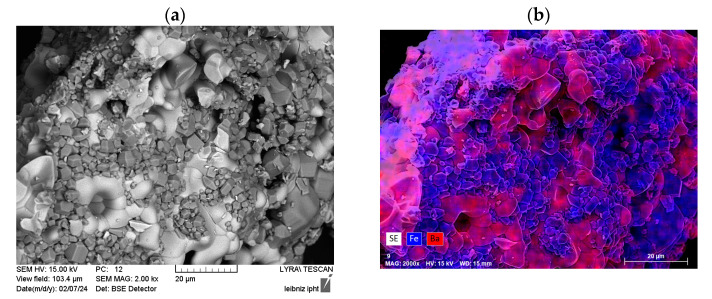
SEM images for sample r = 6.5 (**a**) and corresponding EDX mapping with Fe in blue and Ba in pink (**b**) for the same sample and field of view.

For sample r = 6.5, some bright particles were seen in the SEM images with a different morphology and brighter Z-contrast in the BSE images (see Figure 10). Similarly, sample r = 8 exhibited some particles of brighter Z-contrast as well (picture not shown). EDX analysis confirmed barium-rich material in those smooth-looking particle areas. However, for samples with even higher amounts of the barium-rich BaFe_2_O_4_ phase (r = 2 and r = 5), the SEM images did not exhibit such differently shaped particles.

#### 3.2.2. Morphology and Structural Characterization

**Figure 11:**
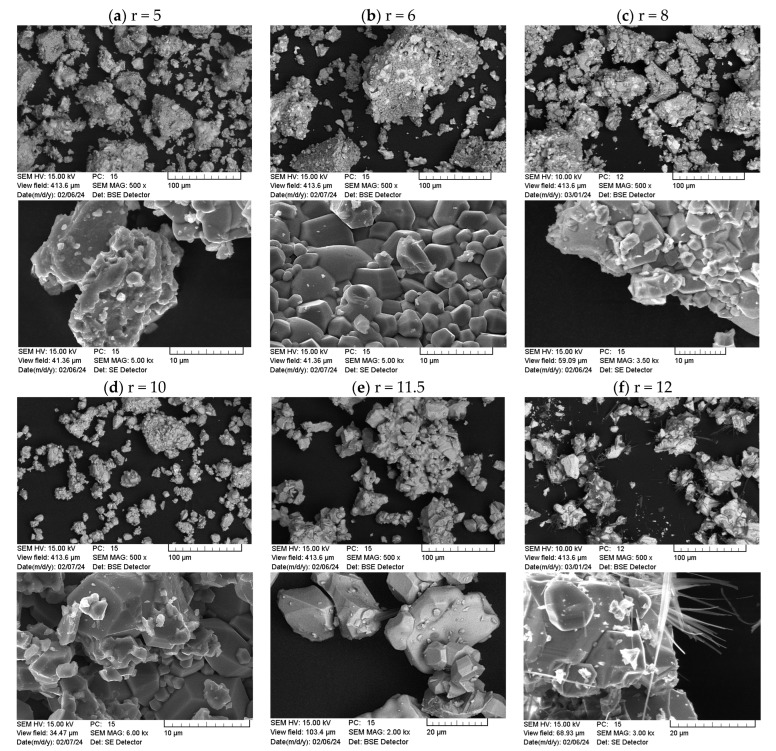
SEM images (SE and BSE mode) of samples with different Fe/Ba ratio (r), given above each column. The upper pictures in each column are acquired with lower magnifications and the lower pictures for each r are acquired with higher magnifications.

In Figure 11, SEM images of samples with r ranging from 5 to 12 can be seen. Again, large agglomerates and a broad size distribution are evident. For samples with a high r of 11.5 and 12, the particles appear larger and the fraction of the smallest particles seen in the samples with a low r vanishes. Sample r = 12 (Figure 11f) exhibited needle- or fiber-like structures in addition to the particles. In the EDX analysis of these needles, a high amount of sodium and oxygen was found as well as a minor contribution of iron. Therefore, we assume some sort of sodium iron oxide as the material forming the needles. In the XRD analysis, very low amounts of 0.5 wt% Fe_12_Na_4_O_20_ were detected for this sample, matching the EDX results regarding the detected sodium. 

**Figure 12:**
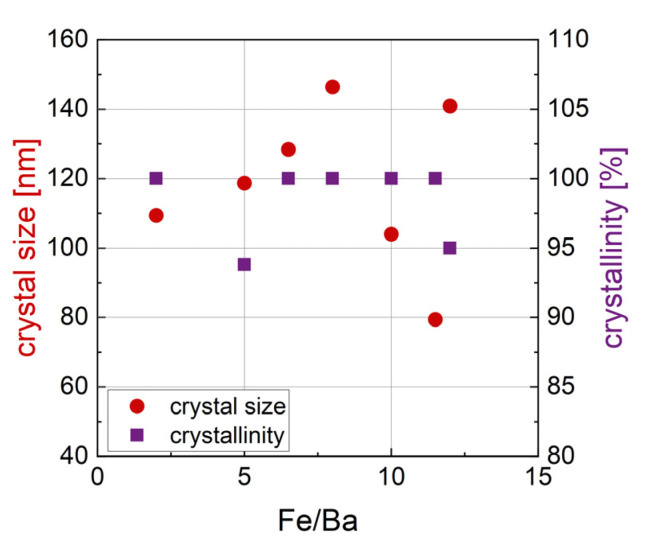
Size (red dots) and crystallinity (purple squares) for BaFe-NP with varying Fe/Ba ratio.

The relation of crystal size and crystallinity with r can be seen in Figure 12. The size and crystallinity do not show clear trends with an increasing Fe/Ba ratio. The size ranges from 79.4 nm to 146.4 nm and appears to be random in relation to the Fe/Ba ratio. Note that the size was calculated by a Williamson–Hall plot of the peaks assigned to the BaFe_12_O_19_ phase; therefore, it only characterizes the size of crystals of this phase. The crystallinity of the sample is high in general, with only two samples, r = 5 and r = 12, exhibiting a crystallinity below 100%, of 93.8% and 95%, respectively.

#### 3.2.3. Magnetic Characterization

**Figure 13:**
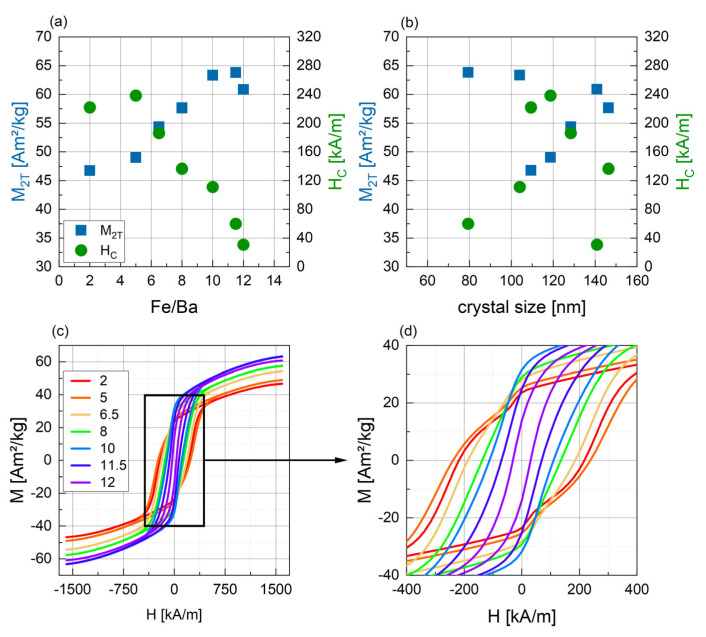
Magnetic parameters M_2T_ (blue) and H_C_ (green) for BaFe-NP synthesized with varying Fe/Ba ratio (**a**) and in correlation with the respective crystal size values (**b**) including linear fits. Related hysteresis curves are shown in (**c**) and magnifications of the low-field region of hysteresis curves are shown in (**d**).

The results of the magnetic characterization can be seen in Figure 13. M_2T_ and H_C_ both show clear trends with the Fe/Ba ratio (Figure 13a). Magnetization increases, reaching a maximum value of 63.82 Am^2^/kg for r = 11.5 and decreasing slightly for the stochiometric ratio of 12. The theoretical maximum saturation magnetization for barium hexaferrite bulk material of 72 Am^2^/kg [20,53] is not reached, which is expected for nanoparticles due to the spin canting effects on the surface. Discussing the magnetization of the samples with varying r, the magnetic properties of BaFe_2_O_4_ found in samples with a low r need to be considered. A low magnetization and only weak ferromagnetic behavior are reported for this phase [33,54,55], which matches the decreased magnetization of the samples with r = 2 to 8. However, the highest amount of BaFe_2_O_4_ was detected for sample r = 5, while the lowest magnetization was measured for sample r = 2. Coercivity is highest for r = 5 (238.4 kA/m) and decreases nearly linearly with increasing r to 30.72 kA/m. The theoretical coercivity for barium hexaferrite is 594 kA/m [20] and values as high as 472 kA/m are reported experimentally [40]. The coercivity of the BaFe_2_O_4_ phase found for samples with a low r is reported to be very inhomogeneous at 413 kA/m [54], 96 kA/m [55], 242 kA/m [56] or 318 kA/m [57], being similar or lower compared to barium hexaferrite. The increase in coercivity with a low r therefore cannot be explained by the coercivity of the monoferrite BaFe_2_O_4_.

Looking at Figure 13b, no clear relation of H_C_ and M_2T_ with the crystal size can be seen. For H_C,_ it might be assumed that intermediate crystal sizes lead to the highest H_C_ values, while H_C_ is lower for smaller and for larger crystal sizes. This might indicate a transition from a single-domain state to a multi-domain state. To confirm this relation, further investigations and more data points are required. As discussed above, a decrease in H_C_ with an increase in crystal size is usually explained by the formation of multi-domain structures. In the SEM images in Figure 11, a fraction of very small particles is seen for samples with a lower r, while the particles in samples with r > 10 appear bigger. This tendency towards the formation of entirely multi-domain particles can explain the decrease in coercivity with a rising Fe/Ba ratio. In summary, by increasing the amount of Ba^2+^ in the synthesis, coercivity can be increased, but a compromise between high M_2T_ and H_C_ needs to be found.

The M(H) curves for the samples with a small r (Figure 13c,d) exhibit a slight drop in magnetization for fields close to zero, which was discussed above for high coercivity samples already. This indicates the formation of two different magnetic fractions of different coercivities in those samples, matching the very heterogeneous size distribution seen in the SEM.

## 4. Conclusions

Within this study, we report the synthesis and characterization of barium hexaferrite nanoparticles of high but tunable coercivity. By varying the synthesis parameters pH, d_cal_ and T_cal_ in the synthesis method used, consisting of a precipitation and a calcination step, T_cal_ was identified as an important factor to control the particles’ magnetic behavior. For a high T_cal_ of 1200 °C, the coercivity of the particles decreased significantly, most likely due to multi-domain formation. The crystallinity of the particles was very high, reaching values of 100% or close to it. By changing the ratio of iron to barium ions, a considerable influence on coercivity and magnetization was evident, with M_2T_ increasing and H_C_ decreasing for an increase in the Fe/Ba ratio from 2 to 12. With this method, coercivities can be achieved in the range of 30 to 239 kA/m. Due to this almost linearly tunable coercivity, our proposed particles can be utilized for heating applications that use a high external magnetic field amplitude. Such high fields necessitate particles of high anisotropy for optimal heat generation, which may, however, not exceed a limit for the coercivity, above which the particles are magnetically too hard for efficient heating. By changing the Fe/Ba ratio in our proposed synthesis, the coercivity can be precisely adapted to the external field. Commonly reported barium hexaferrite particles have a very high coercivity, with values higher than 300 kA/m [37,38,40]. Such high coercivities would necessitate an extremely large external field amplitude for efficient heating. Therefore, by controlling the coercivity of the particles in the manner proposed here, the coercivity can be tuned downwards to values suitable for magnetic heating in the available magnetic fields, while at the same time maintaining a high magnetization. Other particles reported in the literature with coercivities in the range of 100 to 200 kA/m show decreased magnetizations [27,30,36]. When optimizing particles for the external field following the results of our study, the number of particles needed for sufficient heat generation can be minimized, which is beneficial for applications such as lateral flow assays. A small number of particles as markers binding to the assay would be sufficient for heat generation above the detection limit, lowering the sensitivity of the assay. This opens the door to future research on the use of barium hexaferrite particles in extracorporeal magnetic heating applications. For future research, the particles and the synthesis parameters, respectively, should be chosen in accordance with the application and, especially, the field parameters used.

However, the particles appear in the SEM images to be highly agglomerated and rather inhomogeneous in size, which necessitates further research on how to control the particles’ size and agglomeration to enable smaller and monodispersed particles. A first approach was made by adding surfactants like CTAB during precipitation to prevent agglomeration. In addition, ongoing studies will investigate the possibilities of different milling techniques for the control of the particles’ size.

## Figures and Tables

**Table 1:** Summary of the synthesis parameters and related sample names used. For parameter variation, the Fe/Ba ratio r was constant at r = 11.5.

Sample	d_cal_	T_cal_	pH
	[h]	[°C]	
3_800_8	3	800	8
3_1000_8	3	1000	8
3_1200_8	3	1200	8
3_800_10	3	800	10
3_1000_10	3	1000	10
3_1200_10	3	1200	10
3_800_12	3	800	12
3_1000_12	3	1000	12
3_1200_12	3	1200	12
9_800_12	9	800	12
12_800_12	12	800	12
24_800_12	24	800	12
3_800_13	3	800	13
3_1000_13	3	1000	13
3_1200_13	3	1200	13

**Table 2:** Results of the characterization with XRD and VSM for the BaFe-NP with varied synthesis parameters. The phase composition, size and crystallinity, magnetization at 2 T M_2T_ and coercivity H_C_ at room temperature are shown.

d_cal_ [h]	T_cal_ [°C]	pH	Phase 1 [wt%]	Phase 2 [wt%]	Phase 3 [wt%]	Size [nm]	Crystal-Linity [%]	M_2T_ [Am^2^/kg]	H_C_ [kA/m]
3	800	8	BaFe_12_O_19_ (96.8)	α-Fe_2_O_3_ (3.2)		64.3	97.7	60.5	375.6
3	1000	8	BaFe_12_O_19_ (95.9)	α-Fe_2_O_3_ (4.1)		88.8	100	59.3	373.3
3	1200	8	BaFe_12_O_19_ (87.1)	α-Fe_2_O_3_ (10.5)	Ba(OH)_2_ (2.4)	115.3	100	52.5	45.4
3	800	10	BaFe_12_O_19_ (100)			61.2	95.4	56.5	427.0
3	1000	10	BaFe_12_O_19_ (100)			73.8	97.8	58.5	419.6
3	1200	10	BaFe_12_O_19_ (100)			180.8	100	62.0	50.6
3	800	12	BaFe_12_O_19_ (94.6)	BaFe_18_O_27_ (5.4)		78.5	96.7	58.3	418.0
3	1000	12	BaFe_12_O_19_ (100)			98.4	98.6	61.0	386.0
3	1200	12	BaFe_12_O_19_ (99.0)	BaFe_2_O_4_ (1.0)		108.7	100	63.2	70.0
9	800	12	BaFe_12_O_19_ (93.7)	α-Fe_2_O_3_ (6.3)		52	100	58.1	408.1
12	800	12	BaFe_12_O_19_ (99.7)	BaFe_2_O_4_ (0.3)		62.9	100	59.5	408.6
24	800	12	BaFe_12_O_19_ (100)			63.3	96.2	59.0	400.6
3	800	13	BaFe_12_O_19_ (99.3)	FeNaO_2_ (0.7)		47.3	91.7	51.0	441.7
3	1000	13	BaFe_12_O_19_ (100)			240.1	100	55.5	403.2
3	1200	13	BaFe_12_O_19_ (100)			77.4	100	60.0	120.1

**Table 3:** Results of the characterization with XRD and VSM for the BaFe-NP with varied Fe/Ba ratio r. The phase composition, size and crystallinity, magnetization at 2 T M_2T_ and coercivity H_C_ at room temperature are shown.

r = Fe/Ba	Phase 1 [wt%]	Phase 2 [wt%]	Phase 3 [wt%]	Size [nm]	Crystal- Linity [%]	M_2T_ [Am^2^/kg]	H_C_ [kA/m]
2	BaFe_12_O_19_ (80.1)	BaFe_2_O_4_ (19.9)		109.4	100	46.8	221.9
5	BaFe_12_O_19_ (72.5)	BaFe_2_O_4_ (25.5)	Fe_21.4_O_32_ (2.0)	118.7	93.8	49.0	238.4
6.5	BaFe_12_O_19_ (89.0)	BaFe_2_O_4_ (11.0)		128.4	100	54.4	186.2
8	BaFe_12_O_19_ (94.6)	BaFe_2_O_4_ (5.4)		146.4	100	57.7	136.6
10	BaFe_12_O_19_ (93.0)	BaFeO_2.67_ (0.6)	BaFeO_2.89_ (6.4)	104.0	100	63.3	111.1
11.5	BaFe_12_O_19_ (100)			79.4	100	63.8	60.03
12	BaFe_12_O_19_ (99.5)	Fe_12_Na_4_O_20_ (0.5)		140.9	95	60.8	30.72

## Data Availability

Data are available on reasonable request from the corresponding author.
